# Conditional depletion of transcriptional kinases Ctk1 and Bur1 and effects on co-transcriptional spliceosome assembly and pre-mRNA splicing

**DOI:** 10.1080/15476286.2021.1991673

**Published:** 2021-10-27

**Authors:** Isabella E. Maudlin, Jean D. Beggs

**Affiliations:** Wellcome Centre for Cell Biology, School of Biological Sciences, University of Edinburgh, Edinburgh, UK

**Keywords:** Transcription elongation, CTD, serine 2 phosphorylation, pre-mRNA splicing, yeast

## Abstract

From yeast to humans, pre-mRNA splicing occurs mainly co-transcriptionally, with splicing and transcription functionally coupled such that they influence one another. The recruitment model of co-transcriptional splicing proposes that core members of the transcription elongation machinery have the potential to influence co-transcriptional spliceosome assembly and pre-mRNA splicing. Here, we tested whether the transcription elongation kinases Bur1 and Ctk1 affect co-transcriptional spliceosome assembly and pre-mRNA splicing in the budding yeast *Saccharomyces cerevisiae*. In *S. cerevisiae*, Ctk1 is the major kinase that phosphorylates serine 2 of the carboxy-terminal domain of the largest subunit of RNA polymerase II, whilst Bur1 augments the kinase activity of Ctk1 and is the major kinase for elongation factor Spt5. We used the auxin-inducible degron system to conditionally deplete Bur1 and Ctk1 kinases, and investigated the effects on co-transcriptional spliceosome assembly and pre-mRNA splicing. Depletion of Ctk1 effectively reduced phosphorylation of serine 2 of the carboxy-terminal domain but did not impact co-transcriptional spliceosome assembly or pre-mRNA splicing. In striking contrast, depletion of Bur1 did not reduce phosphorylation of serine 2 of the carboxy-terminal domain, but reduced Spt5 phosphorylation and enhanced co-transcriptional spliceosome assembly and pre-mRNA splicing, suggesting a role for this kinase in modulating co-transcriptional splicing.

## Introduction

Pre-mRNA splicing, the process by which introns are removed, relies on a combination of trans-acting factors and short conserved sequences within introns. Trans-acting factors recognize these sequence motifs and position the pre-mRNA for the two transesterification reactions catalysed by the spliceosome. The spliceosome is a large macromolecular complex composed of five small nuclear RNAs (snRNAs) and approximately 300 proteins that fall into two classes: small nuclear ribonucleoprotein particles (snRNPs) – U1, U2, U4, U6 and U5 – and numerous non-snRNP proteins. The five snRNPs assemble in a step-wise manner on a precursor messenger RNA (pre-mRNA) transcript. Firstly, the U1 snRNP binds to the 5ʹ splice site (SS) followed by the U2 snRNP, which binds to the intron branchpoint (BP), forming the pre-spliceosome or A complex. Addition of the pre-assembled tri-snRNP (U4/U6.U5) forms the transient pre-B complex, which is the only stage of spliceosome assembly that contains all five snRNPs assembled on the pre-mRNA substrate. The spliceosome undergoes structural rearrangements during which the U1 snRNP leaves to form the B complex, then U4 snRNP leaves, to generate Bact complex (containing U2, U5 and U6) followed by further rearrangements to form the catalytically activated spliceosome or B* complex, which catalyses the first step of splicing (generating the C complex). This is followed by further structural rearrangements (generating the C* complex) that promote the second catalytic step of splicing (reviewed in [1]).

It is increasingly evident that pre-mRNA splicing occurs co-transcriptionally in many organisms – as RNA polymerase II (RNAPII) transcribes along the gene and before transcription termination [2–15]. Together, the speed of RNAPII and the recruitment of splicing factors by the transcription elongation machinery are thought to be important in regulating the splicing outcome. It is possible that elongation factors that dynamically associate with and/or modify RNAPII to facilitate transcription through the nucleosome barrier influence co-transcriptional splicing. This could occur by elongation factors facilitating the initial recruitment of splicing factors to newly synthesized pre-mRNA substrates, by providing a platform to stabilize their association once recruited, and/or by modulating the speed of RNAPII (reviewed in [16–21]). This study focuses on the potential contribution of two core members of the transcription elongation machinery in *S. cerevisiae*, the kinases Bur1 (CDK9 in mammals) and Ctk1 (CDK12 in mammals), to co-transcriptional spliceosome assembly and pre-mRNA splicing. Both kinases phosphorylate the large unstructured ‘carboxy-terminal domain’ or ‘CTD’ in the largest subunit of RNAPII (Rpb1) that protrudes from the catalytic core. The CTD is a low-complexity domain that is conserved from yeast to humans and comprises heptapeptide repeats with the consensus amino acid sequence YSPTSPS, of which there are 26 repeats in *S. cerevisiae* and 52 in humans (reviewed in [22]). The CTD is likened to a ‘landing pad’ for the recruitment of transcription elongation factors that regulate stages of RNAPII transcription and chromatin modification. Individual residues within the heptad repeats of the CTD are subject to post-translational modifications that dynamically change during the transcription cycle, enabling diverse regulation of these processes (reviewed in [22]). Five of the seven residues (tyrosine 1, serine 2, threonine 4, serine 5 and serine 7) of the CTD heptad repeats are reversibly phosphorylated according to the transcriptional progress of RNAPII along a gene. Phosphorylation makes the CTD less compact and more extended in structure (reviewed in [23]).

Of relevance to the present work is phosphorylation of serine 2 of the CTD repeats, a modification associated with transcription elongation that increases towards the 3ʹ end of genes [24]. Ctk1 is the major, yet non-essential, serine 2 kinase of the CTD of RNAPII in *S. cerevisiae* [25,26]. Bur1 is an essential kinase that was proposed to augment the CTD serine 2 phosphorylation activity of Ctk1 at the 5ʹ ends of genes, in addition to being the major kinase of the conserved C-terminal region (CTR) of Spt5, an essential elongation factor conserved from yeast to humans (DSIF in mammals) ([27–29], reviewed in [30]). Together, Bur1 and Ctk1 had been thought to re-constitute the function of mammalian P-TEFb that in metazoans phosphorylates DSIF and NELF to relieve promoter-proximal pausing by RNAPII, a phenomenon that is conserved in higher eukaryotes [31–33]. However, it is now established that CDK12 is the metazoan homolog of Ctk1 and CDK9 is the metazoan homolog of Bur1 [34]. An orthologue of NELF has not been identified in *S. cerevisiae*, but loss of Bur1 increases RNAPII transcription arrest due to loss of Spt5 phosphorylation [27–29,35–37]. In contrast, loss of Ctk1 does not affect RNAPII elongation rate or processivity in *S. cerevisiae* [27,38,39,40]. Bur1 and Ctk1 associate with elongating RNAPII, with Bur1 recruited to RNAPII phosphorylated at serine 5 of the CTD, whilst Ctk1 kinase activity is stimulated by Bur1 kinase activity [28]. Recently, it was shown that Bur1 and Ctk1 associate with chromatin *via* interaction with nascent RNA [41].

As core components of the transcription elongation machinery, it is conceivable that Bur1 and Ctk1 could influence co-transcriptional pre-mRNA splicing. For example, in mammalian cells, it was shown that mutation of serine 2 of the CTD to alanine reduced recruitment of the U2 snRNA to sites of transcription, and caused defects in pre-mRNA splicing [42]. Further, it was shown that splicing factor U2AF65 interacts directly with the serine 2 phosphorylated CTD of RNAPII [43]. It has also been proposed that, in mammalian cells, pre-mRNA splicing influences the elongation rate of RNAPII *via* P-TEFb [44,45], and that P-TEFb interacts with splicing factor SKIP (Prp45 in *S. cerevisiae*) [46]. Collectively, these studies demonstrate links between serine 2 phosphorylation, P-TEFb and pre-mRNA splicing in mammalian cells. However, in *S. cerevisiae* a mutant RNAPII with a CTD in which the serine 2 residues were mutated to alanine in the first eight repeats did not affect pre-mRNA splicing [8].

It is therefore an open question whether Bur1 or Ctk1 (and phosphorylation of their substrates) affects co-transcriptional spliceosome assembly or pre-mRNA splicing in *S. cerevisiae*. We have investigated this using the auxin-inducible degron (AID) system [47] to conditionally deplete Bur1 or Ctk1. Depletion of Ctk1 resulted in loss of serine 2 phosphorylation of the CTD of RNAPII without affecting co-transcriptional spliceosome assembly or pre-mRNA splicing. In contrast, depletion of Bur1 did not affect serine 2 phosphorylation of the CTD but substantially reduced Spt5 phosphorylation, caused enhanced co-transcriptional recruitment of the U2 and U5 snRNPs and improved co-transcriptional pre-mRNA splicing efficiency.

## Results

### Conditional depletion of Bur1 or Ctk1

**Figure 1.**
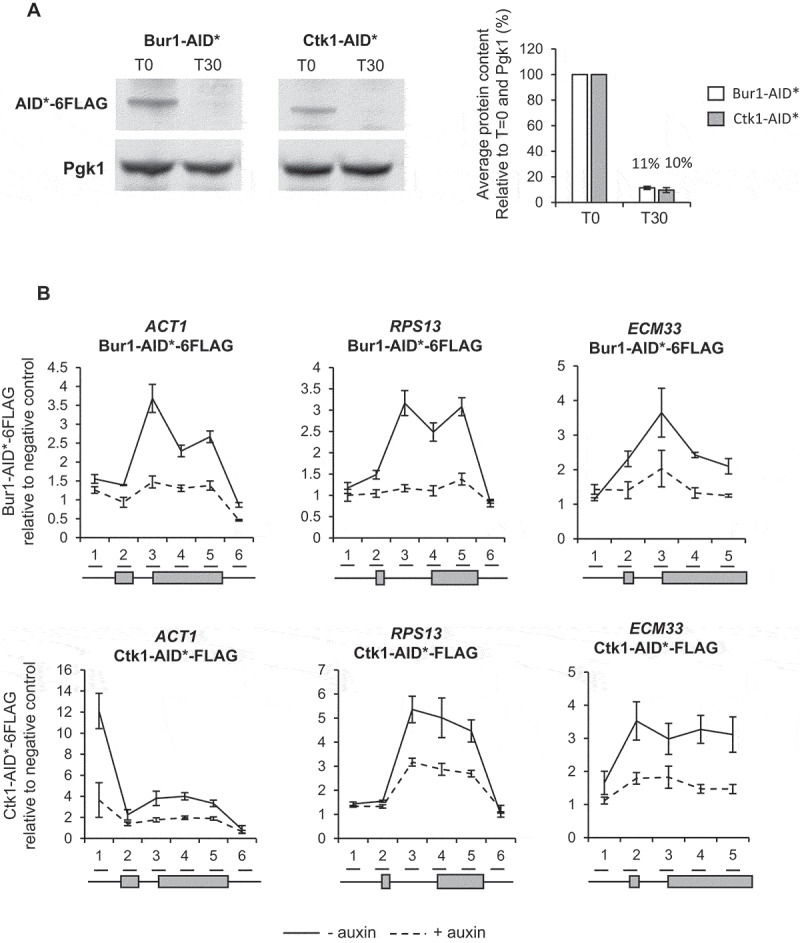
Conditional depletion of Bur1 and Ctk1

To enable conditional depletion of Bur1 or Ctk1 using the AID system, each was C-terminally tagged with the AID* degron and 6 X FLAG epitope tag in strains that constitutively express a Tir1 F-box protein that allows auxin-dependent depletion of degron-tagged proteins [47,48]. In the absence of auxin, the AID* tag does not affect the ability of either Bur1 or Ctk1 to support growth when compared with the untagged parental strain (PADH1-409-TIR1) (Figure S1). Western blotting showed that 30 minutes of auxin treatment reduced the level of Bur1 or Ctk1 on average to 11% and 10% respectively relative to conditions prior to auxin treatment (Figure 1(A)). Chromatin immunoprecipitation and quantitative PCR (ChIP-qPCR) of Bur1 and Ctk1 showed that, in addition to being depleted in whole cell extracts, they were well depleted across chromatin at the intron-containing genes *ACT1, RPS13* and *ECM33* (Figure 1(B)). The intron-containing genes (*ACT1, RPS13* and *ECM33*) used throughout this study were chosen as they are well expressed and are spliced co-transcriptionally [15].

(A) Western blots probed with anti-FLAG (to detect Bur1-AID* and Ctk1-AID*) and anti-Pgk1 as a loading control. Samples were taken before (T0) and 30 minutes (T30) after addition of auxin. Bur1-AID* (white bars) and Ctk1-AID* (grey bars) depletions were quantified and shown as the percentage mean of at least three biological replicates for T30 relative to T0 and normalized to the Pgk1 signal. Error bars = standard error of the mean. (B) Anti-FLAG ChIP followed by qPCR analysis of the intron-containing genes *ACT1, RPS13* and *ECM33* without (-) auxin (solid black line) or (+) 30 minutes after auxin (dashed black line) addition to deplete Bur1-AID* (upper three graphs) or Ctk1-AID* (lower three graphs). The X-axis shows the positions of amplicons used for ChIP-qPCR analysis, with exons represented by grey rectangles. The data are presented as the mean percentage of input relative to a negative control amplicon. Error bars = standard error of the mean. Asterisks show the statistical significance (Student’s unpaired t-test). *P < 0.05, ** *P* < 0.01 and *** *P* < 0.001. Not significant = *P* > 0.05.

### Depletion of Bur1 reduces Spt5 phosphorylation, whereas depletion of Ctk1 reduces serine 2 phosphorylation of the CTD

To determine whether this level of depletion of Bur1 or Ctk1 reduced phosphorylation of their major substrates, western blotting was performed using antibodies specific for the serine 2 phosphorylated form of the CTD of RNAPII or for the phosphorylated form of Spt5 [27]. This showed that, following depletion of Bur1, Spt5 phosphorylation was reduced on average to 18% relative to conditions prior to auxin addition and to total Spt5, but was unaffected by depletion of Ctk1 (Figure 2(A)). On the other hand, following depletion of Ctk1, serine 2 phosphorylation decreased on average to 17% relative to conditions prior to auxin addition and to Rpb3 (Figure 2(B)), whereas it increased, on average, to 120% following depletion of Bur1. As a control, serine 5 phosphorylation of the CTD was analysed, showing an increase in this phosphorylation state to 127% and 136% following depletion of Bur1 or Ctk1, respectively (Figure 2(C)).

**Figure 2.**
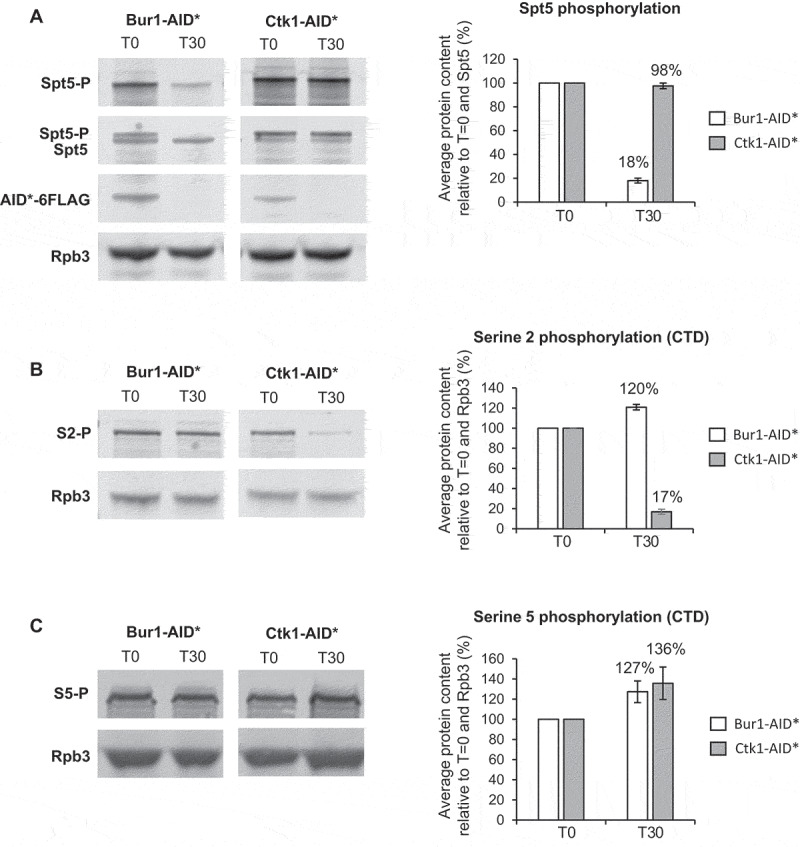
Depletion of Bur1 reduces Spt5 phosphorylation, whereas depletion of Ctk1 reduces serine 2 phosphorylation of the CTD

(A) Western blots probed with anti-phosphoryated Spt5 (Spt5-P), anti-Spt5, anti-FLAG and anti-Rpb3 (RNAPII) as a loading control. Samples were taken before (T0) and 30 minutes (T30) after addition of auxin to deplete Bur1-AID* or Ctk1-AID*. Spt5 phosphorylation levels were quantified after depletion of Bur1-AID* (white bars) and Ctk1-AID* (grey bars) and shown as the percentage mean of 3 biological replicates after 30 minutes of auxin treatment relative to time zero and normalized to Spt5 signal. (B) Western blots probed with anti-phosphorylated serine 2 (S2P) and anti-Rpb3 (RNAPII) as a loading control. Samples were taken before (T0) and 30 minutes (T30) after addition of auxin to deplete Bur1-AID* or Ctk1-AID*. Serine 2 phosphorylation levels were quantified after depletion of Bur1-AID* (white bars) and Ctk1-AID* (grey bars) and shown as the percentage mean of 3 biological replicates after 30 minutes of auxin addition relative to time zero and normalized to Rpb3 signal. (C) Western blot probed with anti-phosphorylated serine 2 (S5P) and anti-Rpb3 (RNAPII) as a loading control. Samples were taken before (T0) and 30 minutes (T30) after addition of auxin to deplete Bur1-AID* or Ctk1-AID*. Serine 5 phosphorylation levels were quantified as described for panel (b). (d,f) Anti-Rpb1 (RNAPII) and (e,g) anti-phosphorylated serine 2 (S2P) ChIP and qPCR across *ACT1, RPS13* and *ECM33* without auxin (- auxin; solid lines) and after 30 minutes of auxin treatment to deplete Bur1-AID* (+ auxin; dashed lines; D, E) or Ctk1-AID* (+ auxin; dashed lines; F, G). The X-axes show the positions of amplicons used for ChIP qPCR analysis – the exons are in grey. The Rpb1 ChIP data are presented as the mean percentage of input relative to the first amplicon of each gene. The S2P ChIP data are relative to Rpb1 occupancy. All data (d–g): mean of at least three biological replicates, error bars = standard error of the mean.

**Figure 2.**
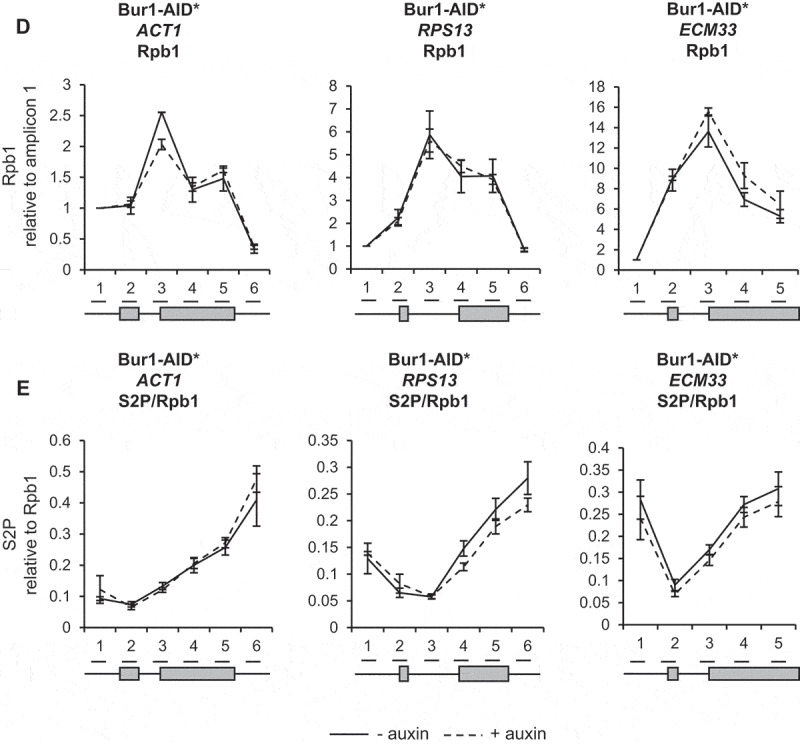
Continued

**Figure 2.**
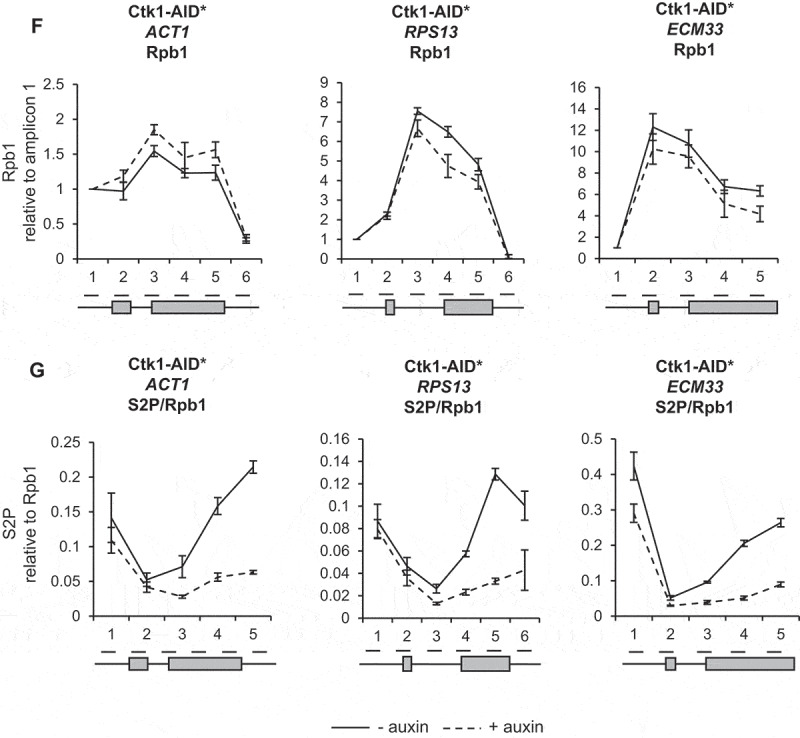
Continued

ChIP analysis using antibodies against total RNAPII (Rpb1) followed by qPCR showed that depletion of Bur1 or Ctk1 for 30 minutes did not significantly affect RNAPII occupancy across the intron-containing genes *ACT1, RPS13* and *ECM33*, relative to conditions prior to depletion (Figure 2(D,F)). ChIP analysis with antibodies specific for phosphorylated serine 2 of the CTD (S2P) showed no significant effect of depleting Bur1 (Figure 2(E)), but depletion of Ctk1 reduced S2P where it is normally highest, at the 3ʹ ends of these genes (Figure 2(G)).

### Depletion of Bur1 causes enhanced co-transcriptional recruitment of the U2 and U5 snRNPs and increases co-transcriptional pre-mRNA splicing efficiency

As splicing factors assemble co-transcriptionally on intron-containing pre-mRNAs, their close-proximity to chromatin enables them to be crosslinked to DNA and analysed by ChIP-qPCR. In this way, the co-transcriptional recruitment of splicing factors and spliceosome assembly can be monitored *in vivo* [7,10,49]. To determine whether depletion of Bur1 in these experimental conditions affects co-transcriptional spliceosome assembly, ChIP-qPCR was performed across the intron-containing genes *ACT1, RPS13* and *ECM33* using antibodies that detect core members of the spliceosome: the U1 snRNP (Prp40), U2 snRNP (Lea1-3HA) or U5 snRNP (Prp8). This analysis showed that U1 snRNP occupancy across these genes was not significantly affected by depletion of Bur1 (Figure 3(A)). In contrast, the U2 snRNP signal was elevated on *ACT1* (at the 3’SS and exon 2) and on *ECM33* (at the 3’SS), relative to conditions prior to depletion (Figure 3(B)). Depletion of Bur1 also caused increased signal for the U5 snRNP on *ACT1* (at exon 2), *RPS13* (3’SS and exon 2) and *ECM33* (exon 2), relative to conditions prior to depletion (Figure 3(C)).

**Figure 3.**
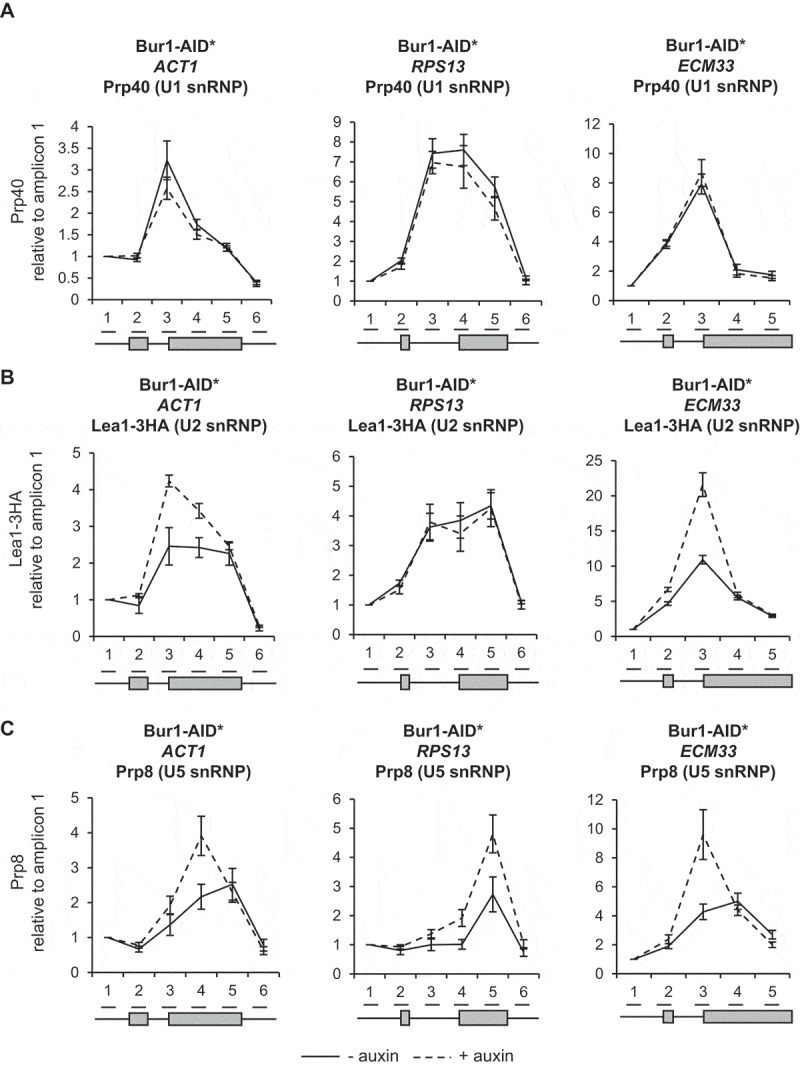
Depletion of Bur1 causes enhanced co-transcriptional recruitment of the U2 and U5 snRNPs and increases co-transcriptional pre-mRNA splicing efficiency

(A) Anti-Prp40 (U1 snRNP) (B) anti-Lea1-3HA (U2 snRNP) and (C) anti-Prp8 (U5 snRNP) ChIP followed by qPCR analysis for *ACT1, RPS13* and *ECM33*, 0 minutes (- auxin; solid lines) and after 30 minutes (+ auxin; dashed lines) of auxin treatment to deplete Bur1-AID*. The X-axes show the positions of amplicons used for ChIP-qPCR analysis – the exons are in grey. The data are presented as the mean percentage of input relative to the first amplicon of each gene. (D) NET-RT-qPCR analysis of *ACT1, RPS13* and *ECM33* transcripts after depletion of Bur1-AID*, normalized to the intronless *ALG9* PolII transcript and time zero (no auxin). Primers used detected pre-mRNA (5’SS or BP and 3’SS), lariat (excised intron or intron-exon 2), exon 2 (ex 2) or mRNA. All data (a–d): mean of at least three biological replicates, error bars = standard error of the mean.

**Figure 3.**
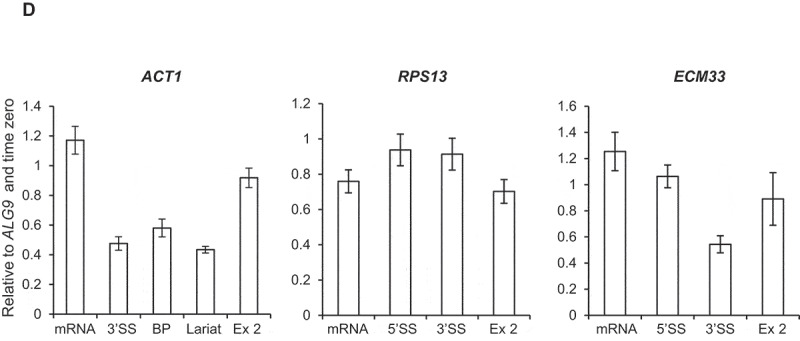
Continued

Next, the effect of Bur1 depletion on co-transcriptional splicing was tested by NET-RT-qPCR whereby a native pull-down of transcriptionally engaged RNAPII is performed followed by

purification of associated nascent RNA transcripts and RT-qPCR [50,51]. For *ACT1* and *ECM33*, this showed an approximately 20% increase in spliced mRNA levels and reduced or unchanged amounts of unspliced or partially spliced transcripts (3’SS, BP and lariat RNA amplicons) associated with RNAPII upon Bur1-AID* depletion, whilst the exon 2 signals did not significantly change (Figure 3(D)). No significant changes were detected in pre-mRNA, spliced mRNA or exon 2 levels of *RPS13* associated with RNAPII upon Bur1-AID* depletion (Figure 3(D)).

### Depletion of Ctk1 does not affect co-transcriptional spliceosome assembly or pre-mRNA splicing

To test whether depletion of Ctk1 and loss of serine 2 phosphorylation of the CTD affected co-transcriptional spliceosome assembly, ChIP-qPCR was performed as described above. This showed no significant change in the signal for U1, U2 or U5 snRNPs following Ctk1 depletion for 30 minutes, relative to conditions prior to auxin addition (Figure 4(A–C)). Therefore, in these conditions, *in vivo* loss of Ctk1 and serine 2 phosphorylation does not affect co-transcriptional spliceosome assembly.

**Figure 4.**
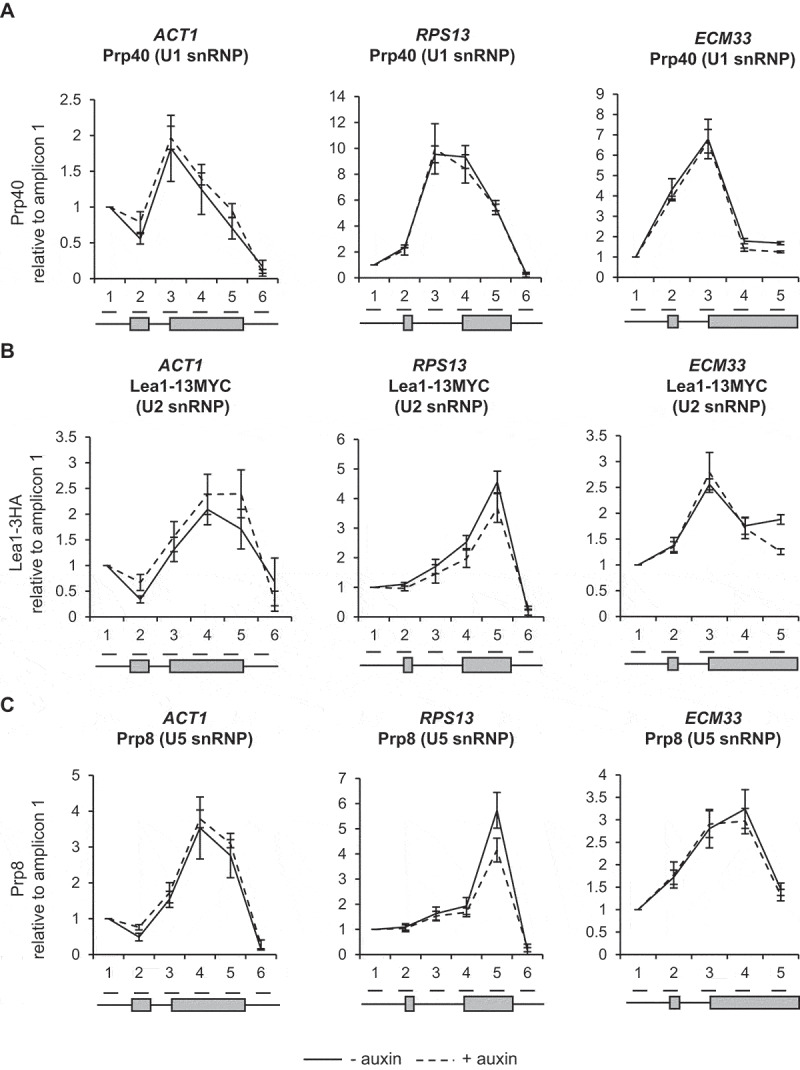
Depletion of Ctk1 does not affect co-transcriptional spliceosome assembly or pre-mRNA splicing

(A) Anti-Prp40 (U1 snRNP) (B) anti-Lea1-13MYC (U2 snRNP) and (C) anti-Prp8 (U5 snRNP) ChIP followed by qPCR analysis for *ACT1, RPS13* and *ECM33*, 0 minutes (- auxin; black) and after 30 minutes (+ auxin; dashed lines) of auxin treatment to deplete Ctk1-AID*. The X-axes show the positions of amplicons used for ChIP qPCR analysis – the exons are in grey. The data are presented as the mean percentage of input relative to the first amplicon of each gene. (D) Splicing RT-qPCR analysis of the intron-containing *ACT1, RPS13* and *ECM33* transcripts in total RNA after depletion of Bur1-AID*, normalized to the *SCR1* PolIII transcript and time zero (no auxin). Primers used detected pre-mRNA (5’SS or BP and 3’SS), lariat (excised intron or intron-exon 2), exon 2 (ex 2) or mRNA. All data (a–d): mean of at least three biological replicates, error bars = standard error of the mean.

**Figure 4.**
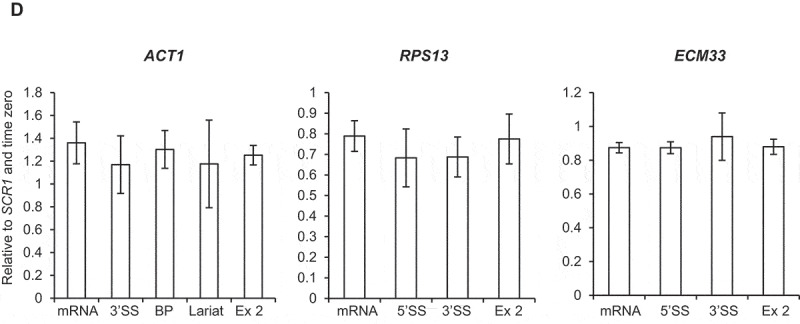
Continued

To test whether depletion of Ctk1 affects pre-mRNA splicing, RT-qPCR was performed on total (steady-state) RNA. No significant accumulation of pre-mRNA species upon depletion of Ctk1 was observed for *ACT1, RPS13* and *ECM33* (Figure 4(D)), meaning depletion of Ctk1 does not result in pre-mRNA splicing defects, which fits with the ChIP-qPCR data, showing no effect on co-transcriptional spliceosome assembly upon Ctk1 depletion (Figure 4(A–C)).

## Discussion

In this study, depletion of Ctk1 was an effective way to reduce serine 2 phosphorylation of the CTD of RNAPII at the 3ʹ ends of genes, without affecting RNAPII occupancy. This result supports Ctk1 being the major serine 2 CTD kinase in *S. cerevisiae* (Figure 2(B)) [25,52]. In contrast, Bur1 depletion did not reduce serine 2 phosphorylation of the CTD (Figure 2(B)). This is in agreement with the report that mutant Bur1 lacking kinase activity does not affect serine 2 phosphorylation of the CTD, as measured by ChIP [35]. However, this is in contrast with reports that deletion of *BUR2* (cyclin of Bur1) and inhibition of Bur1 kinase activity reduce both the overall level of serine 2 phosphorylated RNAPII as measured by western blotting and the amount at the 5ʹ end of the *ARG1* gene measured by ChIP in *S. cerevisiae* [27,28,52,53]. These conflicting observations could reflect the difference between a conditional depletion system and genetic deletions/mutations, or could be due to differences in antibodies used for detection of the phosphorylated CTD of RNAPII, or it may be specific to the genes analysed.

Loss of Ctk1 and, subsequently, of serine 2 phosphorylation of the CTD of RNAPII did not affect co-transcriptional spliceosome assembly or pre-mRNA splicing (Figure 4(A–D)). This is in agreement with the observation that mutant RNAPII with a CTD in which the serine 2 residues were mutated to alanine in the first eight repeats had no effect on splicing in *S. cerevisiae* [8]. However, it contrasts with the report that mutation of serine 2 of the mammalian CTD to alanine reduced recruitment of the U2 snRNA to transcription sites, causing defects in pre-mRNA splicing [42], and that the serine 2 phosphorylated CTD of RNAPII directly recruits splicing factor U2AF65 [43]. These apparently conflicting observations may be explained by differences in the complexity of splicing between *S. cerevisiae* and mammals. Moreover, our finding suggests that Ctk1 does not have substrates within the spliceosome that are important for co-transcriptional spliceosome assembly or splicing in *S. cerevisiae*.

As expected, depletion of Bur1 reduced Spt5 phosphorylation, whereas Ctk1 depletion did not (Figure 2(A)). Interestingly, Bur1 depletion resulted in a significantly increased ChIP signal for the U2 and U5 snRNPs at *ACT1* and *ECM33*, and for the U5 snRNP at *RPS13* (Figure 3(B,C)) that we interpret to represent increased co-transcriptional recruitment of these snRNPs to nascent transcripts. The observation that U1 snRNP signal did not change (Figure 3(A)), indicates that, following Bur1 depletion, U1 associates and then dissociates normally upon B complex formation (as U1 is not in B complex).

It could be argued that the accumulation of the U2 and U5 snRNPs under these conditions might be explained by changes in RNAPII elongation upon depletion of Bur1. In our experimental conditions, depletion of Bur1 did not significantly affect RNAPII occupancy (by ChIP analysis) or RNA production (exon 2 levels of nascent RNA and steady-state RNA) for the genes tested (Figures 2(D) and 3(D)). Further, if RNAPII production was affected, one would expect changes in occupancy of all the snRNPs, whereas the occupancy of the U1 snRNP was unaffected in these conditions. An alternative explanation is that RNAPII could be pausing more frequently, or for longer, allowing more time for spliceosomes to assemble co-transcriptionally on more of the nascent transcripts, thereby explaining better detection of the snRNPs by ChIP. Indeed, it was reported that Bur1 mutants confer sensitivity to 6-azauracil and reduce RNAPII elongation, and that reduced RNAPII occupancy can be detected at the 3ʹ ends of long genes [27–29,35–37,54]. Our conclusion that depletion of Bur1 does not significantly affect RNAPII occupancy on the genes analysed agrees with the report that inhibition of Bur1 kinase activity does not affect RNAPII occupancy or transcription [27], and that deletion of Bur2 has no effect on RNAPII occupancy in *S. cerevisiae* [28]. Nevertheless, the genes analysed here are short, as is typical of genes in *S. cerevisiae*, making small transcriptional changes less easily detectable. As U1 snRNP occupancy is normal upon Bur1 depletion, the increased ChIP signal for the U2 and U5 snRNPs could be explained by a delay to the progression of co-transcriptional spliceosome assembly at some stage after B complex formation (i.e. subsequent to dissociation of U1 snRNP, with B or subsequent complexes accumulating co-transcriptionally).

Alternatively, B complex formation and later steps in the splicing cycle might occur more efficiently in the absence of Bur1, with more splicing occurring co-transcriptionally. Indeed, loss of Bur1 enhanced co-transcriptional splicing of *ACT1* and *ECM33* (assayed by NET-RT-qPCR), pointing to enhanced co-transcriptional assembly of spliceosomes and more efficient splicing in the absence of Bur1. Another possibility, that increased U2 and U5 snRNP signal is due to accumulation of a post-splicing complex, can be ruled out as excised introns do not accumulate (which should be detected by NET-RT-qPCR) and no defects were observed in spliceosome recycling or splicing catalysis upon Bur1 depletion. Conceivably, the gene-specific effects of U2 and U5 snRNP accumulation may be explained by differences between non-ribosomal protein genes (*ACT1* and *ECM33*) and a ribosomal protein gene (*RPS13*). Ribosomal protein transcripts are spliced faster, more efficiently and more co-transcriptionally in comparison to non-ribosomal genes in *S. cerevisiae* [15], therefore, splicing of *RPS13* transcripts may be optimal even in the presence of Bur1, and relatively unaffected by its depletion. A genome-wide analysis would be required to test this hypothesis.

Together, data presented here show that loss of Bur1 directly or indirectly enhances co-transcriptional assembly of spliceosomes at the stage of B complex formation or later, and in this way stimulates co-transcriptional pre-mRNA splicing by a currently unknown mechanism. It is possible that Bur1 could facilitate structural rearrangements in the spliceosome *via* interaction with, or phosphorylation of a splicing factor acting at the pre-B to B transition. We previously demonstrated that Spt5 interacts with spliceosomes and promotes co-transcriptional spliceosome assembly at the stage of formation of the pre-B or B complex [55]. However, our analysis of a phosphomutant of Spt5 showed a much milder effect on spliceosome assembly and splicing than depletion of Bur1 (our unpublished results), suggesting that another, as yet unidentified, target of Bur1 may be involved. As Ctk1 depletion did not affect co-transcriptional spliceosome assembly, the effects observed of Bur1 depletion are not likely to be due to loss of serine 2 phosphorylation of the CTD.

Together, these data highlight a novel point of regulation of co-transcriptional spliceosome assembly in *S. cerevisiae*, functional differences between the budding yeast kinases Bur1 and Ctk1, and differences in the effects of CTD phosphorylation on co-transcriptional spliceosome assembly between yeast and humans.

## Materials and methods

### Yeast strains and growth conditions

The strains used in this work are listed in Table 1. Bur1 and Ctk1 were C-terminally AID*-tagged in a W303 background that constitutively expressed OsTIR (PADH1-409-TIR1) [48]. For tagging, a plasmid containing the AUX/IAA (AID*) followed by a 6 X FLAG tag for immunodetection was used [56]. Lea1 was C-terminally 3-HA-tagged in the Bur1-AID* strain and C-terminally 13-MYC-tagged in the Ctk1-AID* strain.

**Table 1.** Yeast strains used

Name	Genotype	Source
W303	*MATα ade2-1 ura3-1 his3-11,15 trp1-1 leu2-3,112 can1-100*	Beggs lab
PADH1-409-TIR1	*MATα ade2-1 ura3-1 trp1-1 leu2-3,112 can1-100 his3-11,15::PADH1-409-OsTIR1-NatMX*	[48]
PADH1-409-TIR1 Bur1-AID*-6FLAG Lea1-3HA	MATα ade2-1 ura3-1 trp1-1 leu2-3,112 can1-100 his3-11,15::PADH1-409-OsTIR1-NatMX BUR1-AID*-6FLAG-HygMX LEA1-3HA-KAN	This study
W303 Ctk1-AID*-6FLAG Lea1-13-MYC	MATα ade2-1 ura3-1 trp1-1 leu2-3,112 can1-100 his3-11,15::PADH1-409-OsTIR1-NatMX CTK1-AID*-6FLAG-HygMX LEA1-13MYC-KAN	This study
PADH1-409-TIR1 Bur1-AID*-6FLAG Lea1-3HA Rpb3-TAP	MATα ade2-1 ura3-1 trp1-1 leu2-3,112 can1-100 his3-11,15::PADH1-409-OsTIR1-NatMX BUR1-AID*-6FLAG-HygMX LEA1-3HA-KAN RPB3-TAP-HIS	This study

Standard growth conditions were used. For auxin depletion time courses, cells at an OD_600_ of 0.8 were treated with or without 0.75 mM Indole-3-acetic acid (IAA) (auxin) [Acros organics #122160100) for 30 minutes.

### Protein sample preparation and western blotting

Protein was extracted from 5 ml of log-phase culture using a TCA/NaOH precipitation as described in Volland et al. [57]. Equal concentrations of protein (25 μg) were run on a NuPAGE 4–12% Bis-Tris gel (Invitrogen, #NP0323BOX) and transferred to a nitrocellulose membrane (Bio-rad 0.2 µm, #LC2009). Primary antibodies used for western blotting: rat anti‐FLAG (Agilent, #200474), mouse anti‐PGK1 (Abcam, #Ab113687), mouse anti‐Rpb3 (Biolegend, #665004), rat anti-S2P (Chromotek, #3E10), rat anti-S5P (Chromotek, #3E8), Rabbit anti-Spt5-P (#1761, kind gift from Prof. Steven Hahn) [27] and Rabbit anti-Spt5 (kind gift from Prof. Grant Hartzog) [58]. Secondary antibodies used for western blotting: goat anti-mouse IRDye680RD (LI-COR, #926-68070), goat anti-rat IRDye800RD (LI-COR, #926-32219), goat anti-rabbit IRDye800RD (LI-COR, #925-32211). The Odyssey infrared imaging system (LI-COR Bioscience) was used to visualize proteins-of-interest, and signals quantified by the median method of the Odyssey software. For quantitation, data were normalized against the PGK1 signal unless otherwise stated in the figure legend.

### RNA preparation and RT-qPCR

RNA was extracted from 5 ml of log-phase culture using phenol:chloroform, followed by RT-qPCR as described in Alexander et al. [2]. Sequences of primers used for RT-qPCR analysis can be provided if requested. Primers were used for each transcript that detected pre-mRNA (5’ss, 3’SS or the BP], lariat (excised intron lariat or lariat-exon 2), exon 2 and mRNA. Data are normalized to the intronless gene *ALG9* and/or time zero/wild-type conditions, or to *SCR1* RNAPIII transcript and/or time zero/wild-type conditions.

### Chromatin immunoprecipitation (ChIP)

50 ml of log-phase culture was fixed in 1% [w/v) formaldehyde for 10 minutes followed by incubation in 2.5 ml 2.5 M glycine for 5 minutes and harvested by centrifugation. Chromatin was prepared as described in Maudlin and Beggs [55].

Antibodies used for ChIP: rabbit anti-Prp40 (rabbit 11 Eurogentec 2014], rabbit anti-Prp8 (R1703 Final bleed Boon peptide 5/046), Mouse anti-FLAG (Sigma M2 #F1804), rabbit anti-HA (Abcam #AB9110), mouse anti-Rpb1 (Diagenode #C15100055-100), rabbit anti-V5 (Abcam #AB9116), rat anti-S2P (Chromotek, #3E10). Sequences of primers used for qPCR analysis can be provided if requested. ChIP data are presented as percentage of input normalized to the first amplicon of each gene unless otherwise stated.

### Native elongating transcript (NET) purification and RT-qPCR

The NET protocol was used to immunoprecipitate TAP-tagged Rpb3. The protocol used was a modified version of the protocol developed by Churchman and Weissman [51,59] as described in Azlanzadeh et al. [50]. RT-qPCR was performed as described above.

## Supplementary Material

Supplemental Material

## Open access

This research was funded in whole or in part by the Wellcome Trust [104648, 105256]. For Open Access, the author has applied a CC-BY public copyright licence to any author accepted manuscript version arising from this submission.

## References

[1] Wilkinson ME, Charenton C, Nagai K. RNA splicing by the spliceosome. Annu. Rev. Biochem. 2020;89:359–388. 10.1146/annurev-biochem-091719-064225 31794245

[2] Alexander RD, Innocente SA, Barrass JD, et al. Splicing-dependent RNA polymerase pausing in yeast. Mol Cell. 2010;40:582–593. 21095588 10.1016/j.molcel.2010.11.005 PMC3000496

[3] Ameur A, Zaghlool A, Halvardson J, et al. Total RNA sequencing reveals nascent transcription and widespread co-transcriptional splicing in the human brain. Nat Struct Mol Biol. 2011;18:1435–1440. 22056773 10.1038/nsmb.2143

[4] Brugiolo M, Herzel L, Neugebauer KM. Counting on co-transcriptional splicing. F1000Prime Rep. 2013;5:9. Available from: http://www.f1000.com/prime/reports/b/5/9 23638305 10.12703/P5-9 PMC3619158

[5] Carrillo Oesterreich F, Herzel L, Straube K, et al. Splicing of nascent RNA coincides with intron exit from RNA polymerase II. Cell. 2016;165:372–381. 27020755 10.1016/j.cell.2016.02.045 PMC4826323

[6] Carrillo Oesterreich F, Preibisch S, Neugebauer KM. Global analysis of nascent RNA reveals transcriptional pausing in terminal exons. Mol Cell. 2010;40:571–581. 21095587 10.1016/j.molcel.2010.11.004

[7] Görnemann J, Barrandon C, Hujer K, et al. Cotranscriptional spliceosome assembly and splicing are independent of the Prp40p WW domain. Rna. 2011;17:2119–2129. 22020974 10.1261/rna.02646811 PMC3222125

[8] Harlen KM, Trotta KL, Smith EE, et al. Comprehensive RNA polymerase II interactomes reveal distinct and varied roles for each phospho-CTD residue. Cell Rep. 2016;15:2147–2158. 27239037 10.1016/j.celrep.2016.05.010 PMC4966903

[9] Khodor YL, Menet JS, Tolan M, et al. Cotranscriptional splicing efficiency differs dramatically between Drosophila and mouse. Rna. 2012;18:2174–2186. 23097425 10.1261/rna.034090.112 PMC3504670

[10] Kotovic KM, Lockshon D, Boric L, et al. Cotranscriptional recruitment of the U1 snRNP to intron-containing genes in yeast. Mol Cell Biol. 2003;23:5768–5779. 12897147 10.1128/MCB.23.16.5768-5779.2003 PMC166328

[11] Lacadie SA, Rosbash M. Cotranscriptional spliceosome assembly dynamics and the role of U1 snRNA:5′ss base pairing in yeast. Mol Cell. 2005;19:65–75. 15989965 10.1016/j.molcel.2005.05.006

[12] Listerman I, Sapra AK, Neugebauer KM. Cotranscriptional coupling of splicing factor recruitment and precursor messenger RNA splicing in mammalian cells. Nat Struct Mol Biol. 2006;13:815–822. 16921380 10.1038/nsmb1135

[13] Nojima T, Gomes T, Grosso ARF, et al. Mammalian NET-seq reveals genome-wide nascent transcription coupled to RNA processing. Cell. 2015;161:526–540. 25910207 10.1016/j.cell.2015.03.027 PMC4410947

[14] Tilgner H, Knowles DG, Johnson R, et al. Deep sequencing of subcellular RNA fractions shows splicing to be predominantly co-transcriptional in the human genome but inefficient for lncRNAs. Genome Res. 2012;22:1616–1625. 22955974 10.1101/gr.134445.111 PMC3431479

[15] Wallace EWJ, Beggs JD. Extremely fast and incredibly close: cotranscriptional splicing in budding yeast. Rna. 2017;23:601–610. 28153948 10.1261/rna.060830.117 PMC5393171

[16] Bentley DL. Rules of engagement: co-transcriptional recruitment of pre-mRNA processing factors. Curr Opin Cell Biol. 2005;17:251–256. 15901493 10.1016/j.ceb.2005.04.006

[17] Bentley DL. Coupling mRNA processing with transcription in time and space. Nat Rev Genet. 2014;15:163–175. 24514444 10.1038/nrg3662 PMC4304646

[18] Dujardin G, Lafaille C, Petrillo E, et al. Transcriptional elongation and alternative splicing. Biochim Biophys Acta - Genet Regul Mech. 2013;1829:134–140. 10.1016/j.bbagrm.2012.08.005 22975042

[19] Kornblihtt AR, De La Mata M, Fededa JP, et al. Multiple links between transcription and splicing. Rna. 2004;10:1489–1498. 15383674 10.1261/rna.7100104 PMC1370635

[20] Merkhofer EC, Hu P, Johnson TL. Introduction to cotranscriptional RNA splicing. Methods Mol Biol. 2014;1126:83–96. 24549657 10.1007/978-1-62703-980-2_6 PMC4102251

[21] Perales R, Bentley D. ‘Cotranscriptionality’: the transcription elongation complex as a nexus for nuclear transactions. Mol Cell. 2009;36:178–191. 19854129 10.1016/j.molcel.2009.09.018 PMC2770090

[22] Eick D, Geyer M. The RNA polymerase II carboxy-terminal domain (CTD) code. Chem Rev. 2013;113:8456–8490. 23952966 10.1021/cr400071f

[23] Meinhart A, Kamenski T, Hoeppner S, et al. A structural perspective of CTD function. Genes Dev. 2005;19:1401–1415. 15964991 10.1101/gad.1318105

[24] Komarnitsky P, Cho EJ, Buratowski S. Different phosphorylated forms of RNA polymerase II and associated mRNA processing factors during transcription. Genes Dev. 2000;14:2452–2460. 11018013 10.1101/gad.824700 PMC316976

[25] Cho EJ, Kobor MS, Kim M, et al. Opposing effects of Ctk1 kinase and Fcp1 phosphatase at Ser 2 of the RNA polymerase II C-terminal domain. Genes Dev. 2001;15:3319–3329. 11751637 10.1101/gad.935901 PMC312848

[26] Lee JM, Greenleaf AL. CTD kinase large subunit is encoded by CTK1, a gene required for normal growth of Saccharomyces cerevisiae. Gene Expr. 1991;1:149–167. 1820212 PMC5952209

[27] Liu Y, Warfield L, Zhang C, et al. Phosphorylation of the transcription elongation factor Spt5 by yeast Bur1 kinase stimulates recruitment of the PAF complex. Mol Cell Biol. 2009;29:4852–4863. 19581288 10.1128/MCB.00609-09 PMC2725703

[28] Qiu H, Hu C, Hinnebusch AG. Phosphorylation of the Pol II CTD by KIN28 enhances BUR1/BUR2 recruitment and Ser2 CTD phosphorylation near promoters. Mol Cell. 2009;33:752–762. 19328068 10.1016/j.molcel.2009.02.018 PMC2683426

[29] Zhou K, Kuo WHW, Fillingham J, et al. Control of transcriptional elongation and cotranscriptional histone modification by the yeast BUR kinase substrate Spt5. Proc Natl Acad Sci U S A. 2009;106:6956–6961. 19365074 10.1073/pnas.0806302106 PMC2678430

[30] Hartzog GA, Fu J. The Spt4-Spt5 complex: a multi-faceted regulator of transcription elongation. Biochim Biophys Acta - Genet Regul Mech. 2013;1829:105–115. 10.1016/j.bbagrm.2012.08.007 PMC3545043 22982195

[31] Adelman K, Lis JT. Promoter-proximal pausing of RNA polymerase II: emerging roles in metazoans. Nat Rev Genet. 2012;13:720–731. 22986266 10.1038/nrg3293 PMC3552498

[32] Mayer A, Lidschreiber M, Siebert M, et al. Uniform transitions of the general RNA polymerase II transcription complex. Nat Struct Mol Biol. 2010;17:1272–1278. Available from: http://www.nature.com/doifinder/10.1038/nsmb.1903 20818391

[33] Wood A, Shilatifard A. Bur1/Bur2 and the Ctk complex in yeast: the split personality of mammalian P-TEFb. Cell Cycle. 2006;5:1066–1068. 16721054 10.4161/cc.5.10.2769

[34] Bartkowiak B, Liu P, Phatnani HP, et al. CDK12 is a transcription elongation-associated CTD kinase, the metazoan ortholog of yeast Ctk1. Genes Dev. 2010;24:2303–2316. 20952539 10.1101/gad.1968210 PMC2956209

[35] Keogh M-C, Podolny V, Buratowski S. Bur1 kinase is required for efficient transcription elongation by RNA polymerase II. Mol Cell Biol. 2003;23:7005–7018. 12972617 10.1128/MCB.23.19.7005-7018.2003 PMC193923

[36] Lidschreiber M, Leike K, Cramer P. Cap completion and C-terminal repeat domain kinase recruitment underlie the initiation-elongation transition of RNA polymerase II. Mol Cell Biol. 2013;33:3805–3816. 23878398 10.1128/MCB.00361-13 PMC3811861

[37] Rodríguez-Gil A, García-Martínez J, Pelechano V, et al. The distribution of active RNA polymerase II along the transcribed region is gene-specific and controlled by elongation factors. Nucleic Acids Res. 2010;38:4651–4664. 20385590 10.1093/nar/gkq215 PMC2919717

[38] Ahn SH, Kim M, Buratowski S. Phosphorylation of serine 2 within the RNA polymerase II C-terminal domain couples transcription and 3′ End processing. Mol Cell. 2004;13:67–76. 14731395 10.1016/s1097-2765(03)00492-1

[39] Laribee RN, Krogan NJ, Xiao T, et al. BUR kinase selectively regulates H3 K4 trimethylation and H2B ubiquitylation through recruitment of the PAF elongation complex. Curr Biol. 2005;15:1487–1493. 16040246 10.1016/j.cub.2005.07.028

[40] Mason PB, Struhl K. Distinction and relationship between elongation rate and processivity of RNA polymerase II in vivo. Mol. Cell. 2005;17:831–840 15780939 10.1016/j.molcel.2005.02.017

[41] Battaglia S, Lidschreiber M, Baejen C, et al. RNA-dependent chromatin association of transcription elongation factors and pol II CTD kinases. Elife. 2017;6:e25637. 28537551 10.7554/eLife.25637 PMC5457138

[42] Gu B, Eick D, Bensaude O. CTD serine-2 plays a critical role in splicing and termination factor recruitment to RNA polymerase II in vivo. Nucleic Acids Res. 2013;41:1591–1603. 23275552 10.1093/nar/gks1327 PMC3561981

[43] David CJ, Boyne AR, Millhouse SR, et al. The RNA polymerase II C-terminal domain promotes splicing activation through recruitment of a U2AF65-Prp19 complex. Genes Dev. 2011;25:972–982. 21536736 10.1101/gad.2038011 PMC3084030

[44] Fong YW, Zhou Q. Stimulatory effect of splicing factors on transcriptional elongation. Nature. 2001;414:929–933. 11780068 10.1038/414929a

[45] Lin S, Coutinho-Mansfield G, Wang D, et al. The splicing factor SC35 has an active role in transcriptional elongation. Nat Struct Mol Biol. 2008;15:819–826. Available from: http://www.nature.com/doifinder/10.1038/nsmb.1461 18641664 PMC2574591

[46] Brès V, Gomes N, Pickle L, et al. A human splicing factor, SKIP, associates with P-TEFb and enhances transcription elongation by HIV-1 Tat. Genes Dev. 2005;19:1211–1226. 15905409 10.1101/gad.1291705 PMC1132007

[47] Nishimura K, Fukagawa T, Takisawa H, et al. An auxin-based degron system for the rapid depletion of proteins in nonplant cells. Nat Methods. 2009;6:917–922. 19915560 10.1038/nmeth.1401

[48] Mendoza-Ochoa GI, Barrass JD, Terlouw BR, et al. A fast and tuneable auxin-inducible degron for depletion of target proteins in budding yeast. Yeast. 2019;36:75–81. 30375036 10.1002/yea.3362 PMC6587778

[49] Tardiff DF, Rosbash M. Arrested yeast splicing complexes indicate stepwise snRNP recruitment during in vivo spliceosome assembly. Rna. 2006;12:968–979. 16618970 10.1261/rna.50506 PMC1464846

[50] Aslanzadeh V, Huang Y, Sanguinetti G, et al. Transcription rate strongly affects splicing fidelity and cotranscriptionality in budding yeast. Genome Res. 2018;28:203–213. 29254943 10.1101/gr.225615.117 PMC5793784

[51] Churchman LS, Weissman JS. Nascent transcript sequencing visualizes transcription at nucleotide resolution. Nature. 2011;469:368–373. 21248844 10.1038/nature09652 PMC3880149

[52] Chun Y, Joo YJ, Suh H, et al. Selective kinase inhibition shows that Bur1 (Cdk9) phosphorylates the Rpb1 linker in vivo. Mol Cell Biol. 2019;39. DOI:10.1128/MCB.00602-18 PMC6639251 31085683

[53] Dronamraju R, Strahl BD. A feed forward circuit comprising Spt6, Ctk1 and PAF regulates Pol II CTD phosphorylation and transcription elongation. Nucleic Acids Res. 2014;42:870–881. 24163256 10.1093/nar/gkt1003 PMC3902893

[54] Murray S, Udupa R, Yao S, et al. Phosphorylation of the RNA polymerase II carboxy-terminal domain by the Bur1 cyclin-dependent kinase. Mol Cell Biol. 2001;21:4089–4096. 11390638 10.1128/MCB.21.13.4089-4096.2001 PMC87070

[55] Maudlin IE, Beggs JD. Spt5 modulates cotranscriptional spliceosome assembly in Saccharomyces cerevisiae. RNA. 2019;25:1298–1310. 31289129 10.1261/rna.070425.119 PMC6800482

[56] Morawska M, Ulrich HD. An expanded tool kit for the auxin-inducible degron system in budding yeast. Yeast. 2013;30:341–351. 23836714 10.1002/yea.2967 PMC4171812

[57] Volland C, Urban-Grimal D, Géraud G, et al. Endocytosis and degradation of the yeast uracil permease under adverse conditions. J Biol Chem. 1994;269:9833–9841. 8144575

[58] Hartzog GA, Wada T, Handa H, et al. Evidence that Spt4, Spt5, and Spt6 control transcription elongation by RNA polymerase II in Saccharomyces cerevisiae. Genes Dev. 1998;12:357–369. 9450930 10.1101/gad.12.3.357 PMC316481

[59] Churchman LS, Weissman JS. Native elongating transcript sequencing (NET-seq). Curr. Protoc. Mol. Biol. 2012;1:1–17 10.1002/0471142727.mb0414s98 22470065

