# Supplementary material for: Conditional depletion of transcriptional kinases Ctk1 and Bur1 and effects on co-transcriptional spliceosome assembly and pre-mRNA splicing
Source: RNA Biol. 2021 Oct 27;18(Suppl 2):782–93. doi: 10.1080/15476286.2021.1991673 (PMC8782173; doi:10.1080/15476286.2021.1991673)
Supplement: Supplemental Material [file KRNB_A_1991673_SM6610.zip › Supplementary Figure S1 legend.docx]

**Supplementary Figure S1.** Growth (OD600) of the control parental strain (W303-OsTIR; no AID*-tagged proteins; black) and (A) Bur1-AID*-tagged or (B) Ctk1-AID*-tagged strains (grey) were measured over time. Mean of three biological replicates.
