# Supplementary material for: Conditional depletion of transcriptional kinases Ctk1 and Bur1 and effects on co-transcriptional spliceosome assembly and pre-mRNA splicing
Source: RNA Biol. 2021 Oct 27;18(Suppl 2):782–93. doi: 10.1080/15476286.2021.1991673 (PMC8782173; doi:10.1080/15476286.2021.1991673)
Supplement: Supplemental Material [file KRNB_A_1991673_SM6610.zip › supplementary figure S1.pptx]

## Slide 1
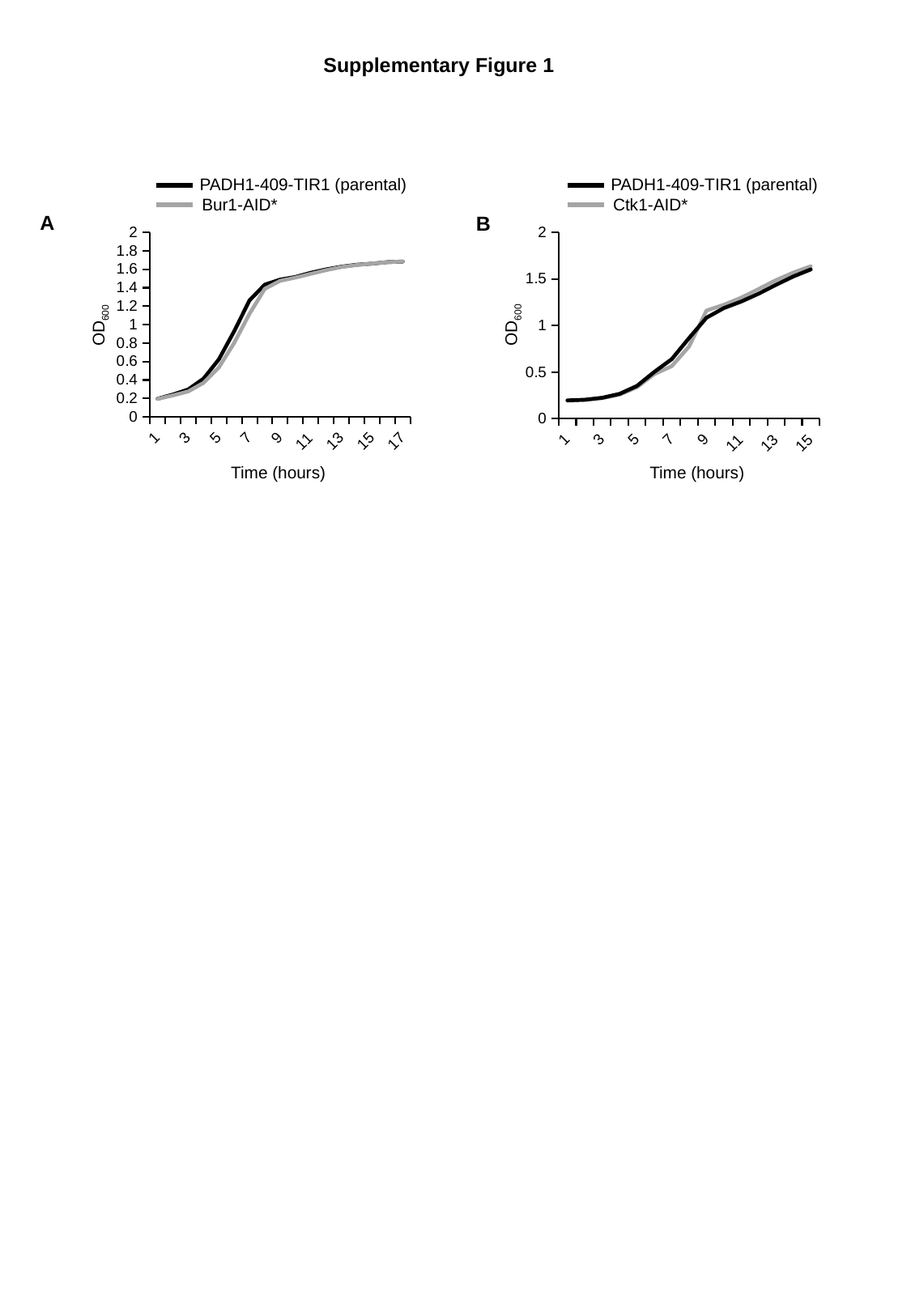

Supplementary Figure 1
OD600
OD600
PADH1-409-TIR1 (parental)
PADH1-409-TIR1 (parental)
Bur1-AID*
Ctk1-AID*
A
B
### Chart
| Category | | |
|---|---|---|
### Chart
| Category | | |
|---|---|---|Time (hours)
Time (hours)
